# Photo-Realistic Image Dehazing and Verifying Networks via Complementary Adversarial Learning

**DOI:** 10.3390/s21186182

**Published:** 2021-09-15

**Authors:** Joongchol Shin, Joonki Paik

**Affiliations:** Department of Image, Chung-Ang University, Seoul 06974, Korea; jcshin@ipis.cau.ac.kr

**Keywords:** dehazing, GAN, CNN

## Abstract

Physical model-based dehazing methods cannot, in general, avoid environmental variables and undesired artifacts such as non-collected illuminance, halo and saturation since it is difficult to accurately estimate the amount of the illuminance, light transmission and airlight. Furthermore, the haze model estimation process requires very high computational complexity. To solve this problem by directly estimating the radiance of the haze images, we present a novel dehazing and verifying network (DVNet). In the dehazing procedure, we enhanced the clean images by using a correction network (CNet), which uses the ground truth to learn the haze network. Haze images are then restored through a haze network (HNet). Furthermore, a verifying method verifies the error of both CNet and HNet using a self-supervised learning method. Finally, the proposed complementary adversarial learning method can produce results more naturally. Note that the proposed discriminator and generators (HNet & CNet) can be learned via an unpaired dataset. Overall, the proposed DVNet can generate a better dehazed result than state-of-the-art approaches under various hazy conditions. Experimental results show that the DVNet outperforms state-of-the-art dehazing methods in most cases.

## 1. Introduction

In outdoor environments, acquired images lose important information such as contrast and salient edges because the particles attenuate the visible light. This degradation is referred to as hazy degradation, which distorts both spatial and color features and decreases visibility of the outdoor object. If the hazy degradation is not restored, we cannot expect a good performance of main image processing or image analysis methods such as object detection, image matching, and imaging systems [1,2,3,4], to name a few. Therefore, the common goal of dehazing algorithms is to enhance the edge and contrast while suppressing intensity or color saturation. To the best of the authors’ knowledge, Middleton and Edgar were the first to employ a physical haze model for the dehazing problem [5].

To generate the haze-free image using the physical model, atmospheric light and the corresponding transmission should be estimated. However, an accurate estimation of the atmospheric light and transmission map generally requires additional information, such as a pair of polarized images, multiple images under different weather conditions, distance maps, or user interactions [6,7,8,9]. For that reason, many state-of-the-art approaches try to find a better method to estimate the atmospheric light and the transmission map based on reasonable assumptions [10,11,12,13]. He et al. proposed a dark channel prior (DCP)-based haze removal method [14]. They assumed that pixels in the local patch of a clear image have at least one dark pixel. The DCP method works well in most regions that satisfy the DCP assumption, but fails in a white object region. Berman et al. estimated the transmission map using haze-line prior assumption that the pixel coordinates in the color space tend to become closer to the atmospheric light in a hazy image [15]. To find the lower bound of a haze-line, they used the 500 representative colors. While the Berman’s approach enhances color contrast, it is impossible to find representative colors in a severely degraded image by haze or fog. Shin et al. optimized the transmission estimation process using both radiance and reflectance components [16].

Recently, convolutional neural networks (CNN) are being applied not only to image classification, but also to variety of low-level image processing applications [17,18,19,20]. The CNN-based dehazing methods were also proposed in the literature to overcome the limitation of the transmission map estimation using a single image. Cai et al. estimated the transmission to restore a haze image using a DehazeNet [21]. Cai’s method falls in the end-to-end supervised learning approach using synthetic haze and clean patches. To overcome the limitation of haze feature estimation, Ren et al. presented a multi-scale CNN [22]. They also proposed a learning method using the pairs of the simulated haze image and true transmission [23].

To increase the training accuracy, Li et al. combined two CNN modules of the transmission and atmospheric light estimation via all-in-one dehazing network (AODNet) [24].

Zhang et al. proposed a densely connected pyramid dehazing network (DCPDN) optimized by a conditional adversarial learning method [25,26].

The depth information can be incorporated into the transmission estimation process using a supervised learning method. However, it is hard to reflect other quantities such as attenuation, atmospheric light, and illuminance at once because it is difficult to collect the data including the depth, attenuation, airlight, and ideal illuminance maps.

**Figure 1:**
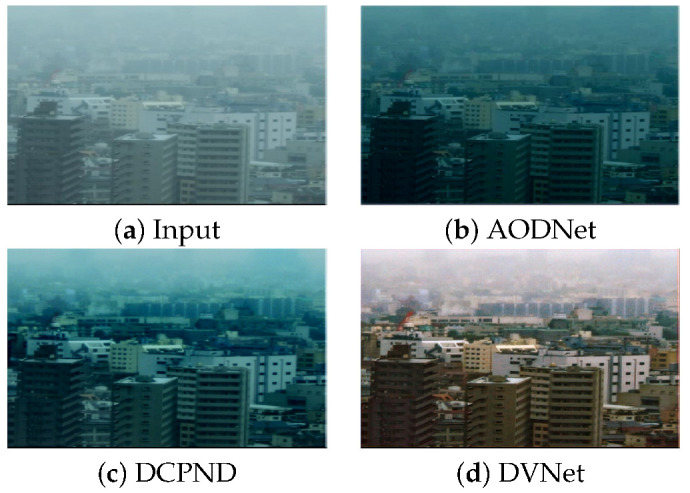
An analysis of limitation of CNN based methods: (**a**) One of real-haze images, (**b**) dehazed result using AODNet, (**c**) dehazed result using DCPDN, and (**d**) dehazed result using our DVNet. Note that the proposed method can restore the most naturally looking image by removing real-haze based on the direct estimation of the radiance map.

For example, Figure 1a shows a real-haze image provided by [27]. This type of haze in Figure 1a is different from what we have simulated, and degraded by multiple factors including the color attenuation, unbalanced light source and scattered light. Therefore, CNN-based estimation can not adaptively remove this real-haze as shown Figure 1b,c.

To overcome the dependency, a radiance estimation method can be applied to the dehazing process. Ren et al. estimated the haze-free radiance by using a mult-scale convolutional neural network and simulated haze dataset [22]. The mult-scale convolutional neural network can stably remove the simulated haze. Chen et al. estimated a physical haze model-based radiance image using a dilated convolution [18] and adaptive normalization [28]. It can approximate the DCP or non-local dehazing operators using low computational complexity. This radiance estimation method can accurately estimate the dehazed result without additional estimation steps, but it may generate the amplified noise and dehazing artifacts. To approach fusion method, Ren et al. removed the haze using derived inputs and gated fusion network [29], Shin et al. proposed the triple convolutional networks including dehazing, enhancement, and concatenating subnetworks to enhance the contrast without dehazing artifacts [30]. However, the separated subnetworks result in increasing computational complexity. To solve this problem, this paper presents a new dehazing and verifying network (DVNet). The proposed DVNet does not need the subnetworks in the prediction procedure. Instead, only correction subnetwork is used for the training process, and evaluates the dehazing error in the output using a complementary adversarial learning. Different from the transmission estimation-based method, the proposed DVNet successfully removed the real-haze without the noise, halo, or other undesired artifacts with low computational complexity. Since the proposed method can use more enhanced ground truth images, our DVNet can be effectively learned by using absolute-mean error and perceptual loss functions. Furthermore, our verifying network simultaneously estimates and reduces the error of the resulting images via self supervised learning and least square adversarial network. Therefore, experimental results show that the proposed DVNet outperforms existing state-of-the-art approaches in the sense of both robustness to various haze environment and computational efficiency. This paper is organized as follows: Section 2 summarized related works, and Section 3, respectively, describes the proposed DVNet and the corresponding training method. After summarizing experimental results in Section 4, we conclude the paper with some discussions in Section 5.

## 2. Related Works

A clear image is degraded by the physical haze model as [5]

(1) $$x^{C}\left(p\right)=t\left(p\right)J^{C}\left(p\right)+\left\{1-t(p)\right\}A^{C}\;\;\mathrm{for}\;\;C\in \left\{R,G,B\right\},$$

where *J* represents a haze-free, clean image, *x* the hazy, degraded version, *p* the two-dimensional pixel coordinate, *t* the light transmission map, and *A* the spatially-invariant atmospheric light. Superscripts in *x*, *J*, and *A* represent a color channel, and the transmission t(p) is independent of the color channel. To solve this equation, physical haze model-based methods estimate the major components such as *t* and *A* based on a proper assumptions. Recently, several deep learning techniques can make this formula solvable without estimating *t* or *A* estimations. Therefore, this section introduces various deep learning-based dehazing approaches.

### 2.1. Physical Haze Model-Based Dehazing

He et al. applied the dark channel prior (DCP) to estimate the transmission as [14]

(2) $$t_{DCP}\left(p\right)=1-\min_{q\in N(p)}\left\{\min_{C\in \{R,G,B\}}\frac{x^{C}\left(q\right)}{A^{C}}\right\},$$

where *q* is the 2D pixel coordinate in a local patch region around *p*, denoted as N(p), in which the transmission is assumed to be constant. Berman et al. estimated the non-local (NL) transmission map using the geometric haze feature as [15]

(3) $$t_{NL}\left(p\right)=\frac{∥x(p)-A∥}{∥J(p)-A∥}.$$

To solve for the feature in (3), Berman et al. used 500 representative colors and approximated the denominator using the *k*-nearest neighbor (k-NN) algorithm [31]. To minimize the dehazing artifacts such as noise and halo in the estimated transmission, either soft matting or weighted least squares [32,33] algorithm can be used as a regularization function. Shin et al. estimate the transmission by minimizing the radiance-reflectance combined cost as [16]

(4) $$\underset{t_{RRO},d_{J}}{\arg\min}\left({∥d_{J}t_{RRO}-d_{I}∥}_{2}^{2}\right),$$

where $d_{I},d_{J}$ are the difference map between the atmospheric light and prior-images such as input and roughly restored input.

### 2.2. Radiance-Based Dehazing

Given *N* pairs of haze-free and its hazy version patches, CNN-based dehazing methods commonly train the network by minimizing the loss function as

(5) $$L_{CNN}\left(\Theta \right)=\frac{1}{N}\sum_{i=1}^{N}∥F\left(x_{i}^{P};\Theta \right)-J_{i}^{P}∥,$$

where $J_{i}^{P}$ and $x_{i}^{P}$ represent the *i*-th training patches of the haze-free and hazy images, respectively. Θ is a set of network parameters including weights and biases, and F(·) is the output of the network given an input hazy image patch and the set of parameters [28,34].

### 2.3. Adversarial Learning

To reduce the divergence between the generated and real images, the adversarial loss can be defined as [26,35,36,37]

(6) $$\arg\min_{\left\{G_{J}\right\}}\max_{\left\{D\right\}}L\left\{1-D(G_{J}\left(I\right))\right\}+L\left\{D(J)\right\},$$

where $G_{J}$ is the haze-free generator, *D* is a discriminator to discriminate a real or fake class, and L{·} denotes a sigmoid cross entropy operator. This adversarial learning can generate a haze-free image that is closer to the clean image.

## 3. Proposed Method

To remove haze, we present a new dehazing and verifying networks using dilated convolution layers and generative adversarial network. Deep learning-based dehazing methods require a serious of procedures including: Generation of dataset, configuration of a deep learning model, and training the model. In this section, we describe the data generation method in Section 3.1, the network architecture and learning functions of both correction and haze nets are given in Section 3.2 and Section 3.3. Section 3.4 presents the proposed training approaches including the verifying network and complementary adversarial learning.

### 3.1. Data Generation

To generate the pairs of the haze and clean images, we first generate the initial dehazed image from the input hazy image using a physical haze model given in (1). Let I(p) be the input hazy image, and $\hat{t}\left(p\right)$ the estimated transmission using either (3) or (4), the initial clean image is computed as

(7) $$I_{D}\left(p\right)=A+\frac{I_{in}\left(p\right)-A}{\hat{t}\left(p\right)}.$$

**Figure 2:**
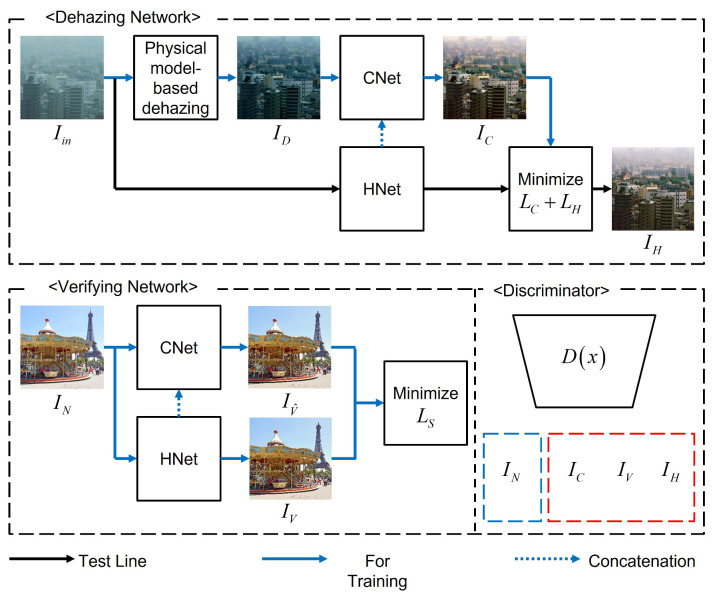
The architecture of the proposed DVNet. Note that the blue and black arrows are used for the training the DVNet. In the prediction procedure, only black arrows are efficiently applied.

Since (7) gives an one-step, closed-form estimation, the training pairs of the hazy and haze-free images can be easily created. In this paper, we used the result of the non-local dehazing (NL) and radiance-reflectance optimization method (RRO) given in (3) and (4) to generate the initial dehazed images. In addition, haze simulated images such as NYU-depth data [23] can also be used to generate $I_{D}$ and $I_{in}$ pair based on physical haze-model. Overall, the generated data $I_{D}$ is used to input data of the correction network as shown in Figure 2. In the dehazing procedure, the input haze images are resoted by the haze network, which is learned by the corrected images. The verifying network imitates the natural images using self supervised learning, and the discriminator classifies the real or fake class between the natural image and generated images to reduce the statistical divergence.

### 3.2. Correction-Network (CNet)

We propose a correction network (CNet) to enhance the initial dehazed images by correcting both color and intensity values. To restore the missing information, we concatenate features of the haze network (HNet) using the dilated convolution and adaptive normalization [18,28] as

(8) $${{\hat{f}}_{i}}^{k}=g\left\{{\overset{⌢}{A}}^{k}{\left({\overset{⌢}{b}}_{i}^{k}+\sum_{j}{{\overset{⌢}{f}}_{j}}^{k-1}{*}_{r_{k}}{\overset{⌢}{h}}_{i,j}\right)}^{k}\right\},$$

where ${{\hat{f}}_{i}}^{k}$ and ${b_{i}}^{k}$, respectively, represent the *i*-th feature map and bias in the *k*-th layer, and ${{\overset{⌢}{h}}_{i,j}}^{k}$ is the kernels to obtain the *i*-th feature map using the feature maps extracted in the *k* − 1st layer, ${\overset{⌢}{f}}^{k-1}$. The operator “*r_k_” represents the dilated convolution using the rate of the *k*-th layer, *r_k_*. The dilated convolution can quickly perform filtering in a wide receptive field without changing the scale. *g* is a leaky rectified linear unit (LReLU) [38] function defined as

(9) $$\begin{array}{c} g\left(x\right)=\max\left(\frac{x}{5},x\right). \end{array}$$

**Figure 3:**
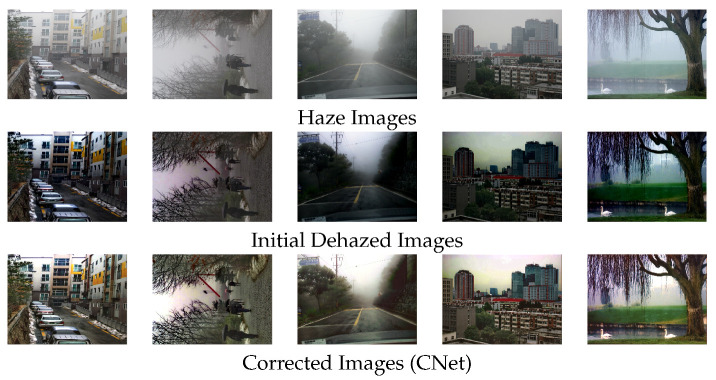
Performance of the proposed CNet. The proposed CNet can adaptively correct the intensity and contrast via HNet features and adaptive normalization. The HNet uses this corrected data as ground truth for complementary learning.

${\overset{⌢}{A}}^{k}$(·) represents the adaptive normalization (AN) function in the *k*-th layer as

(10) $${\overset{⌢}{A}}^{k}\left(x\right)={\overset{⌢}{\alpha }}_{k}x+{\overset{⌢}{\beta }}_{k}BN\left(x\right),$$

where BN(·) denotes the batch normalization function [39], ${\overset{⌢}{\alpha }}_{k}$ and ${\overset{⌢}{\beta }}_{k}$ are the trainable parameters to control the relative portion of the batch normalization function. The adaptive normalization approach given in (10) can provide an enhanced restoration results [28]. In (8), ${\overset{⌢}{f}}^{k-1}$ is concatenated as

(11) $${\overset{⌢}{f}}^{k-1}=\;concat\left[{\hat{f}}^{k-1},f^{k-1}\right],$$

where concat is a feature concatenation operator [40], *f* is the feature map in a HNet that will be described in Section 3.3. This connection plays an important role in coordinating the learning direction. For example, if the CNet is incorrectly learned without the upward connections, the HNet is also learned with different images and such erroneous cycles are repeated. To correctly propagate the learning direction, we concatenate the feature maps of the HNet to the upward feature maps of the CNet. Top of Figure 2 shows the CNet and the proposed upward connection scheme. In addition, the parameters of CNet can be optimized by self-supervised learning using the perceptual loss [41], and it can be defined by VGG16 network [42] which is pretrained using ImageNet data [43]. The perceptual loss in the CNet is referred to as correction loss, which is defined as

(12) $$L_{C}=\frac{1}{N}\sum_{i=1}^{N}\left\{{∥F\left({I_{D}}_{i}\right)-F\left({I_{C}}_{i}\right)∥}_{2}^{2}+{∥{I_{D}}_{i}-{I_{C}}_{i}∥}_{1}+\lambda {∥\nabla {I_{C}}_{i}∥}_{1}\right\},$$

where *N* represents the batch size, $I_{C}$ the output of the C-Net, and *F* returns the feature maps of the VGG16 network model. We used relu1-2, relu2-2, relu3-3 and relu4-3 features in the VGG16. λ is a parameter to regularize $\ell_{1}$-norm of the gradient. This self-supervised CNet can correct color, intensity, and saturation in real-hazy dataset [27] as shown in Figure 3.

### 3.3. Haze-Network (HNet)

The HNet plays an important role in enhancing the degraded images. In addition, an efficient design of the H-Net can significantly reduce the processing time. For that reason, the HNet uses the dilated convolution and adaptive normalization [18,28] as,

(13) $${f_{i}}^{k}=g\left\{A^{k}\left(b_{i}^{k}+\sum_{j}{f_{j}}^{k-1}{*}_{r_{s}}{h_{i,j}}^{k}\right)\right\},$$

where ${f_{i}}^{k}$ is a feature map of the H-Net in the *k*-th layer. *b*, *h*, and $A^{k}\left(\cdot \right)$, respectively, represent the bias, kernel and adaptive normalization operator. Since the HNet is learned using the results of the CNet, its result can also be corrected in an adaptive manner. The HNet can be optimized by minimizing the haze loss as:

(14) $$L_{H}=\frac{1}{N}\sum_{i=1}^{N}\left\{\begin{array}{c} {∥F\left({I_{D}}_{i}\right)-F\left({I_{H}}_{i}\right)∥}_{2}^{2}+{∥{I_{C}}_{i}-{I_{H}}_{i}∥}_{1}+\lambda {∥\nabla {I_{H}}_{i}∥}_{1} \end{array}\right\},$$

where ${I_{H}}_{i}$ is the output of the HNet.

### 3.4. Verifying Network

**Figure 4:**
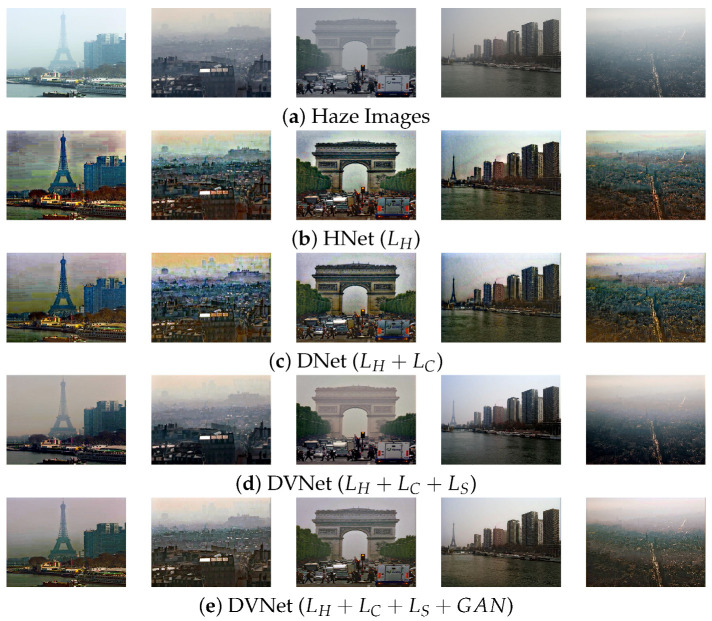
Performance of the proposed DVNet using several challenging example of haze images.

To make the outputs of the dehazing network (HNet, CNet) look more natural, we verify the errors, such as noise and halo artifact, using self-supervised learning with clean data [44]. The verifying loss of the self-supervised learning is defined as

(15) $$L_{S}=\frac{1}{N}\sum_{i=1}^{N}\left\{\begin{array}{c} {∥2F\left({I_{N}}_{i}\right)-\left(F\left({I_{V}}_{i}\right)+F\left({I_{\hat{V}}}_{i}\right)\right)∥}_{2}^{2}\\ +{∥2{I_{N}}_{i}-\left({I_{V}}_{i}+{I_{\hat{V}}}_{i}\right)∥}_{1}+\lambda {∥\nabla {I_{V}}_{i}+\nabla {I_{\hat{V}}}_{i}∥}_{1} \end{array}\right\},$$

where ${I_{N}}_{i}$, ${I_{\hat{V}}}_{i}$, and ${I_{V}}_{i}$, respectively, represent the clean image, results of the CNet, and HNet. Note that the self-supervised terms are designed by considering the errors, which means that the pixels and features in output images of both CNet and HNet are closed to the real natural images when the input images are ideally clean [30]. If input images are the clean images, the ideal haze model should generate the same natural images as in the left-bottom of Figure 2. Therefore this self supervised loss should be separately applied to optimize the networks as Algorithms 1 and 2. In this context, the self-supervised learning based on the loss in (15) using a clean image can minimize the dehazing artifacts as shown in Figure 4d. Futhermore, to reduce the statistical divergence between the generated and real images, the proposed DVNet can be optimized based on the least square adversarial cost [36]

(16) $$\begin{array}{cc} \min_{D}V\left(D\right)= & \\ & E_{I_{N}∼P_{D}\left(I_{N}\right)}\left\{{\left(D\left(I_{N}\right)-1\right)}^{2}\right\}+E_{I_{in}∼P_{G}\left(I_{in}\right)}\left\{{\left(D\left(HNet\left(I_{in}\right)\right)\right)}^{2}\right\}\\ & +E_{I_{D}∼P_{G}\left(I_{D}\right)}\left\{{\left(D\left(CNet\left(I_{D}\right)\right)\right)}^{2}\right\}+E_{I_{N}∼P_{G}\left(I_{N}\right)}\left\{{\left(D\left(HNet\left(I_{N}\right)\right)\right)}^{2}\right\}, \end{array}$$

and

(17) $$\begin{array}{cc} \min_{G}V\left(G\right)= & E_{I_{in}∼P_{G}\left(I_{in}\right)}\left\{{\left(D\left(HNet\left(I_{in}\right)\right)-1\right)}^{2}\right\}\\ & +E_{I_{D}∼P_{G}\left(I_{D}\right)}\left\{{\left(D\left(CNet\left(I_{D}\right)\right)-1\right)}^{2}\right\}\\ & +E_{I_{N}∼P_{G}\left(I_{N}\right)}\left\{{\left(D\left(HNet\left(I_{N}\right)\right)-1\right)}^{2}\right\}, \end{array}$$

where *D* is a convolutional neural net based dicriminator as shown in right-bottom of Figure 2, which returns a probablity value of the input image $I_{*}$ using a binary softmax algorithm. *G* is the generative networks including HNet and CNet. The input data of the discriminator is the ideally natural data $I_{N}$, and the random noise is replaced to real-haze image $I_{in}$, the initial dehazed image $I_{D}$, and natural image $I_{N}$ to engage our HNet and CNet.

In this adversarial learning method, the proposed network can be learned to reduce the probability divergence between the clean image $I_{N}$ and the result of the proposed network ($I_{H},I_{C},I_{V}$) using unfair images. To implement the adversarial cost, we will describe about the optimal parameters in Appendix A.

Therefore, the resulting images ($I_{H},I_{C},I_{V}$) can be improved as the visibility is similar to the clean images ($I_{N}$). Figure 4e shows the performance of the proposed DVNet. More specifically, the resulting images in Figure 4 show that our DVNet can better enhance the hazy images [45] in the sense of both details and contrast without the undesired dehazing artifacts.

**Algorithm 1:** Training procedures of the proposed DVNet**Input:**$I_{in}$, $I_{D}$, $I_{N}$ **Output:**$w_{d}$ **for** iteration from 1 to 15 K do  1: [features, $I_{H}$] = HNet($I_{in}$, $w_{d}$)  2: $I_{C}$ = CNet($I_{D}$, features, $w_{g}$)  3: $I_{V},I_{\hat{V}}$ = VNet($I_{N}$, $w_{d}$, $w_{g}$)  4: $P_{*}$ = Discriminator($I_{C}$, $I_{V}$, $I_{H}$, $I_{N}$, $w_{adv}$)  5: update model by minimizing (14) + (12)  6: update model by minimizing (15)  7: update model by minimizing (16)  8: update model by minimizing (17) **end for**


**Algorithm 2:** Testing procedures of the proposed DVNet**Input:**$I_{in}$, $w_{d}$ **Output:**$I_{H}$  1: $I_{H}$ = HNet($I_{in}$, $w_{d}$)

### 3.5. Implementation

For the implementation, we split our method into the training and testing procedures. The training procedure consists of eight steps: (i) Feature extraction using HNet, (ii) feature concatenation using the CNet and generation of the corrected clean image, (iii) error verification using the same network architecture and natural image [44], (iv) differentiation of the real and fake images using discriminator, (v) minimizing (14) + (12), (vi) minimizing (15), (vii) maximizing and minimizing adversarial costs V(D) and V(G), (viii) repeat the above seven steps until the optimal CNN weights are obtained. The test procedure is simpler than the training procedure, and applies the optimal HNet to remove haze. Table 1 shows the pseudo-code of training and testing procedures of the proposed method. In Table 2 and Table 3, the parameters of the proposed DVNet and discriminator are given for the implementation. To optimize the cost functions, we used an adaptive moment estimation (ADAM) optimization algorithm proposed by [46]. Learning rate values of the DNet and VNet were, respectively, set to $1\times 10^{4}$ and $4\times 10^{4}$. We used 500 real-haze images from the dataset provided by [27], which are engaged to the DVNet with high quality images from NITRE 2017 dataset [44]. Initial clean images were created using the NL, RRO, and NYU-depth data [15,16,23] using five hundred training images. We trained the proposed DVNet 10,000 times. Table 1 shows conventions for the important variables and parameters for the implementation.

## 4. Experimental Results

For the experiment, we selected three benchmark datasets of size 512 × 512 including I-Haze, O-Haze, and 100 real hazy images [27,47,48,49]. Especially for the comparative experiment, we tested existing dehazing methods including: Haze-line prior-based nonlocal dehazing method (NL), densely connected pyramid dehazing net (DCPDN), radiance-reflectance optimization based dehazing (RRO), the region-based haze image enhancement method by using triple convolution network(TCN) [15,16,25,30]. Both NL and RRO were implemented in Matlab 2016b and tested on i7 CPU equipped with 64 GB of RAM. On the other hand, DCPDN, TCN and the proposed method were tested using NVIDIA RTX 2080ti graphics processing unit (GPU) and implemented in Python version 3.6 and Tensorflow. This section includes similarity evaluation in Section 4.1, visual quality evaluation in Section 4.2, and ablation study in Section 4.3.

### 4.1. Similarity Evaluation

For the similarity evaluation, we used three benchmarking datasets including: I-Haze (30), and O-Haze (45) [47,48].

**Figure 5:**
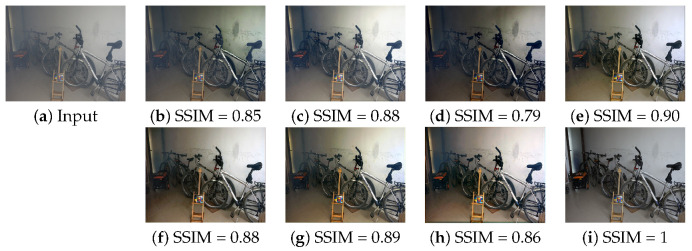
Comparison of dehazed image using I-Haze: (**a**) Haze input, (**b**) NL, (**c**) DCPDN, (**d**) RRO, (**e**) TCN, (**f**) DVNet-NL, (**g**) DVNet-RRO, (**h**) DVNet-NYU, and (**i**) Ground Truth.

**Figure 6:**
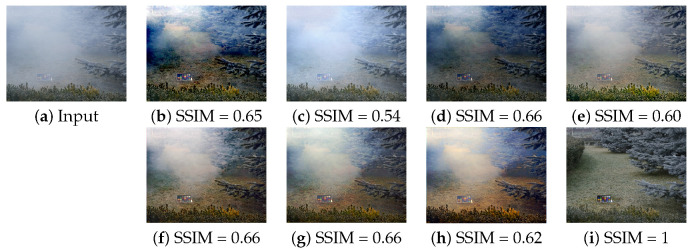
Comparison of dehazed image using O-Haze: (**a**) Haze input, (**b**) NL, (**c**) DCPDN, (**d**) RRO, (**e**) TCN, (**f**) DVNet-NL, (**g**) DVNet-RRO, (**h**) DVNet-NYU, and (**i**) Ground Truth.

For the quantitative evaluation, we measured the peak signal to noise ratio (PSNR), structural similarity index measure (SSIM), and CIE color difference formula 2000 (CIED) [50,51] as shown in Figure 5 and Figure 6 and Table 4, where the best and second best scores are, respectively, shown with blue and cyan colored text. The proposed DVNet is trained by non-local dehazing or radiacne-reflectance optimization-based restoration results or NYU-depth dataset based haze-clean pair.

Both DVNet-RRO and DVNet-NL outperform than state-of-the-art approaches in term of both SSIM, and CIED in I-Haze dataset, which has the ideal illumination because each image was acquired in the indoor environment. However, the performance of DVNet-NYU was slightly lower than TCN-RRO in term of PSNR and SSIM because simulated dataset can not reflect various environments such as airlight and illuminance. It means that the DVNet-NYU can generate intensity saturation as shown in Figure 5h.

Since adaptive normalization used in the TCN and our DVNet stretches the intensity, both DVNet and TCN can change the background color. Therefore, the PSNR of the DVNet-RRO is similar to that of TCN. Note that the DVNet does not only remove the haze but also change the illumination. So the resulting image has a different illuminance from the ground truth image. For that reason, the DVNets and TCN produce a lower similarity in the O-Haze dataset than the NL and RRO approaches.

However the DVNet-RRO performs better than other CNN-based methods such as DCPDN and TCN in term of SSIM.

### 4.2. Visual Qaulity Assessment

**Figure 7:**
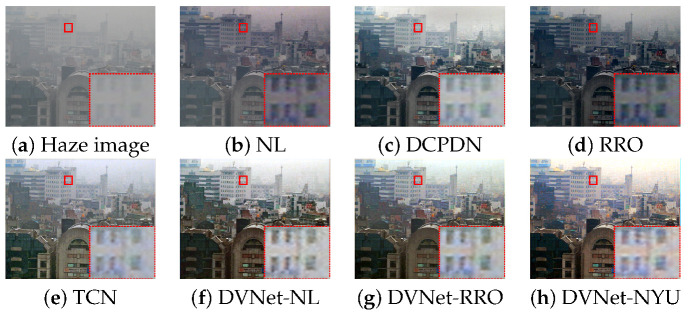
Dehazing results using real-world dataset: The red desh-shaped box denotes a zoomed region in the red box.

**Figure 8:**
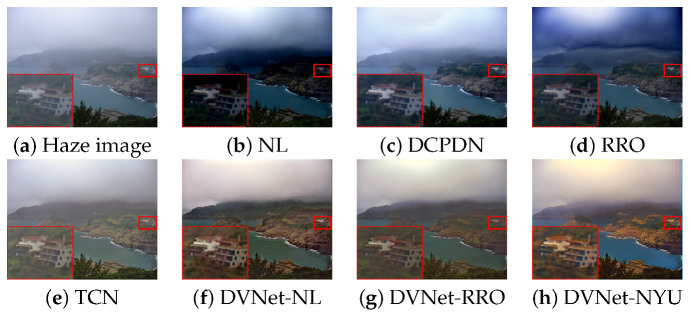
Dehazing results using real-world dataset: The red desh-shaped box denotes a zoomed region in the red box.

To verify the performance of the DVNets in the real haze conditions, we used 100-FADE test sets provided by [27]. For the objective evaluation, we select no-reference measures including: Contrast to noise ratio (CNR), natural image quality evaluation (NIQE), entropy to evaluate amount of information in a single image such as intensity distribution, and intensity saturation [27,52,53]. A high-quality image has high CNR and entropy values, whereas it should have a low NIQE and saturation values for stable enhancement. The average scores of the proposed DVNet-NL are higher than those of state-of-the-art approaches in terms of the CNR and saturation as shown in Table 5. The ranking of the DVNet-NYU was the best score in terms of CNR, entropy, and NIQE. However, due to highly saturated pixels, the color of resultant image of DVNet-NYU can be distorted as shown in Figure 7h. Note that the DVNet-NL has high score in terms of the NIQE with a very small difference from the first NL. The DVNet-RRO also has a similar score in term of NIQE compared with RRO. However, the saturation score of the DVNets are lower than NL and RRO because our DVNets verifies the errors of the NL, RRO, and NYU-depth dataset. In summary, the proposed DVNet can successfully remove various types of haze in various environment [27] as shown in Figure 7 and Figure 8.

### 4.3. Additional Study

To demonstrate the effect the proposed contributions, we conducted the additional studies using the I-Haze and O-Haze datasets. We also used version of the DVNet-NL for the ablation study. In Table 6, HNet and CNet represent the baseline of the proposed dehazing network, DVNet the optimized version of the proposed method with the natural image and self-supervised learning, GAN the optimized version of the proposed method using the proposed adversarial learning method.

Note that the combined HNet and CNet model without VNet returns only similar images to those of physical model-based dehazing method, which also imitates the error such as noise and saturation. Our DNet (HNet+CNet) can reduce the intensity distortion caused by initial dehazed image $I_{D}$. The SSIM values the DVNet increased at the cost of a slight PSNR reduction. This means that our verifying process can prevent the noise and halo at the cost of slightly reduced dehazing performance. However, since the proposed adversarial network complements the dehazing performance, the PSNR values outperform the vanilla DVNet. In addition, Table 7 shows the processing time of the proposed DVNet with various image sizes. In evaluation procedure, the proposed DVNets only use a single network(HNet). Therefore, the DVNets can more reduce the computational time over 5–10 times than the TCN and DCPDN, which have several subnetworks.

## 5. Conclusions

To estimate a high-quality, clean radiance image without the dehazing artifacts, we proposed a novel dehazing network followed by a verifying network, which generates the radiance images to verify the dehazing errors. To estimate an ideally clean image pair, we concatenate feature maps using adaptive normalization and upward connections from the HNet to the CNet. In addition, an unpaired natural image and the discriminator can help minimizing the noise and dehazing artifacts without the performance degradation. The DVNet can be adaptively remove the haze without addtional estimation processes. Therefore, the proposed approach can efficiently remove various types of haze with low conputational complexity. More specifically, three experiments were conducted to verify the performance of the DVNet and the effect of the individual contributions. As a result, the DVNet can provide high-quality dehazing results under various types of haze environments. However, the DVNet may depend on the based training data. In the future work, we plan to combine the DVNet with the data augmentation method, and expand it to video dehazing.

## Figures and Tables

**Table 1:** Conventions of image types.

$I_{in}$	input haze image
$I_{C}$	result of the C-Net
$I_{H}$	generated dehazed image using H-Net
$I_{N}$	natural images for the VNet
$I_{V}$	output of the VNet
$I_{D}$	inintial dehazed image using NL or RRO or NYU

**Table 2:** Details of the proposed DVNet, where Conv denotes a convolution operator, K3 is kernel size of 3 × 3, R is dilation rate, I input channel, O output channel, AN Adaptive normalization, and lrelu is leaky relu.

HNet	CNet
Input $I_{in}$, $I_{N}$	Input $I_{D}$, $I_{N}$
Conv(K3, R1, I3, O24), AN, lrelu	Conv(K3, R1, I3, O24), AN, lrelu
Concat	
Conv(K3, R1, I24, O24), AN, lrelu	Conv(K3, R1, I48, O24), AN, lrelu
Concat	
Conv(K3, R1, I24, O24), AN, lrelu	Conv(K3, R1, I48, O24), AN, lrelu
Concat	
Conv(K3, R2, I24, O24), AN, lrelu	Conv(K3, R2, I48, O24), AN, lrelu
Concat	
Conv(K3, R4, I24, O24), AN, lrelu	Conv(K3, R4, I48, O24), AN, lrelu
Concat	
Conv(K3, R8, I24, O24), AN, lrelu	Conv(K3, R8, I48, O24), AN, lrelu
Concat	
Conv(K3, R16, I24, O24), AN, lrelu	Conv(K3, R16, I48, O24), AN, lrelu
Concat	
Conv(K3, R1, I24, O24), AN, lrelu	Conv(K3, R1, I48, O24), AN, lrelu
Concat	
Conv(K3, R1, I24, O3)	Conv(K3, R1, I48, O3)
Output $I_{H}$, $I_{V}$	Output $I_{C}$, $I_{\hat{V}}$

**Table 3:** Details of the proposed Discriminator, where Conv denotes a convolution operator, K3 is kernel size of 3 × 3, R is dilation rate, I input channel, O output channel, BN Batch normalization, and lrelu is leaky relu.

Discriminator
Input $I_{N}$, $I_{H}$, $I_{V}$, $I_{C}$
Conv (K3, R1, I3, O64), BN, lrelu
Conv (K3, R1, I64, O128), BN, lrelu
Conv (K3, R1, I128, O256), BN, lrelu
Conv (K3, R1, I256, O512), BN, lrelu
FC (I8192, O100), BN, lrelu
FC (I100, O2), Softmax

**Table 4:** Comparison with state-of-the-art dehazing method using various benchmark dataset, where blue and cyan colored numbers are the best and secondly best scores.

-	I-Haze			O-Haze		
Method	PSNR	SSIM	CIED	PSNR	SSIM	CIED
NL [15]	16.00	0.7686	14.2	16.76	0.7842	16.61
DCPDN [25]	14.76	0.7758	15.76	13.20	0.7449	23.79
RRO [16]	14.96	0.7668	15.51	17.23	0.7813	16.51
TCN [30]	17.15	0.7921	14.04	15.47	0.7629	17.04
DVNet-NL	16.76	0.7985	13.62	15.18	0.7657	16.93
DVNet-RRO	17.08	0.8019	13.67	15.21	0.7707	17.31
DVNet-NYU	16.97	0.7907	13.81	15.03	0.7568	18.16

**Table 5:** Visual quality evaluation using CNR, Entropy, NIQE, and saturation, where blue and cyan colored numbers are the best and secondly best scores.

Method	Input	NL	DCPDN	RRO	TCN	DVNet-NL	DVNet-RRO	DVNet-NYU
CNR	129.41	149.03	138.27	148.16	148.16	154.29	147.56	151.06
Entropy	7.02	6.95	7.32	7.16	7.44	7.50	7.50	7.62
NIQE	19.31	18.53	18.88	18.63	19.21	18.57	18.69	18.52
Saturation	0.79	8.22%	3.66%	3.02%	1.33%	1.29%	1.84%	2.34%

**Table 6:** Ablation Study, where **bold** numbers are best scores.

Ablation Study				I-Haze		O-Haze	
HNet	CNet	DVNet	GAN	PSNR	SSIM	PSNR	SSIM
O	X	X	X	15.91	0.6944	14.97	0.6799
O	O	X	X	16.38	0.6964	**15.37**	0.6776
O	O	O	X	16.28	0.7904	14.50	0.7519
O	O	O	O	**16.76**	**0.7985**	15.18	**0.7657**

**Table 7:** Processing time (s) according to image size.

Width & Height Size	256	512	768	1024
DVNet (gpu)	0.005	0.018	0.039	0.065
TCN (gpu)	0.01	0.05	0.18	0.74
DCPDN (gpu)	-	0.05	-	-
RRO (cpu)	0.71	2.42	4.91	8.30
NL (cpu)	3.13	3.71	4.71	6.80

## Data Availability

Not applicable.

## Appendix A. Optimal Parameters

In proposed method, the least square adversarial cost functions are defined as [36]

(A1) $$\begin{array}{cc} \max_{D}V\left(D\right)= & E_{I_{N}∼P_{D}\left(I_{N}\right)}\left\{{\left(D\left(I_{N}\right)-b\right)}^{2}\right\}+E_{I_{in}∼P_{G}\left(I_{in}\right)}\left\{{\left(D\left(HNet\left(I_{in}\right)\right)-a\right)}^{2}\right\}\\ & +E_{I_{D}∼P_{G}\left(I_{D}\right)}\left\{{\left(D\left(CNet\left(I_{D}\right)\right)-a\right)}^{2}\right\}\\ & +E_{I_{N}∼P_{G}\left(I_{N}\right)}\left\{{\left(D\left(HNet\left(I_{N}\right)\right)-a\right)}^{2}\right\}, \end{array}$$

and

(A2) $$\begin{array}{cc} \min_{G}V\left(G\right)= & E_{I_{in}∼P_{G}\left(I_{in}\right)}\left\{{\left(D\left(HNet\left(I_{in}\right)\right)-c\right)}^{2}\right\}\\ & +E_{I_{D}∼P_{G}\left(I_{D}\right)}\left\{{\left(D\left(CNet\left(I_{D}\right)\right)-c\right)}^{2}\right\}\\ & +E_{I_{N}∼P_{G}\left(I_{N}\right)}\left\{{\left(D\left(HNet\left(I_{N}\right)\right)-c\right)}^{2}\right\}. \end{array}$$

To expand the adversarial cost, (A2) can be modified as

(A3) $$\begin{array}{cc} \min_{G}V\left(G\right)= & E_{I_{N}∼P_{D}\left(I_{N}\right)}\left\{{\left(D\left(I_{N}\right)-c\right)}^{2}\right\}+E_{I_{in}∼P_{G}\left(I_{in}\right)}\left\{{\left(D\left(HNet\left(I_{in}\right)\right)-c\right)}^{2}\right\}\\ & +E_{I_{D}∼P_{G}\left(I_{D}\right)}\left\{{\left(D\left(CNet\left(I_{C}\right)\right)-c\right)}^{2}\right\}\\ & +E_{I_{N}∼P_{G}\left(I_{N}\right)}\left\{{\left(D\left(HNet\left(I_{N}\right)\right)-c\right)}^{2}\right\}, \end{array}$$

where *D* returns the probability values via the discriminator using the soft-max algorithm, and *G* represents the proposed generator model including H and CNet. To find the optimal point of the discriminator, V(D) in (A1) can be expressed as

(A4) $$\begin{array}{cc} V\left(D\right)= & E_{x∼P_{D}\left(I_{N}\right)}\left\{{\left(D\left(x\right)-b\right)}^{2}\right\}+E_{x∼P_{G}\left(I_{in}\right)}\left\{{\left(D\left(x\right)-a\right)}^{2}\right\}\\ & +E_{x∼P_{G}\left(I_{D}\right)}\left\{{\left(D\left(x\right)-a\right)}^{2}\right\}+E_{x∼P_{G}\left(I_{N}\right)}\left\{{\left(D\left(x\right)-a\right)}^{2}\right\}\\ & =\frac{1}{2}\int_{x}P_{D}\left(x\right){\left(D\left(x\right)-b\right)}^{2}+\left(3P_{G}\left(x\right)\right){\left(D\left(x\right)-a\right)}^{2}. \end{array}$$

The optimal point of the discriminator $D^{*}$ can be obtained when its partial derivative with respect to *D* is equal to zero, such as

(A5) $$\frac{\partial }{\partial D}\left\{P_{D}\left(x\right){\left(D\left(x\right)-b\right)}^{2}+\left(3P_{G}\right){\left(D\left(x\right)-a\right)}^{2}\right\}=0.$$

Therefore, the optimal point $D^{*}$ can be defined as

(A6) $$D^{*}\left(x\right)=\frac{bP_{D}\left(I_{N}\right)+a3P_{G}}{P_{D}\left(I_{N}\right)+3P_{G}},$$

which can be simplified by defining the real and fake distributions, respectively, denoted as $P_{1}=P_{D}$ and $P_{2}=3P_{G}$,

(A7) $$D^{*}\left(x\right)=\frac{bP_{1}+aP_{2}}{P_{1}+P_{2}},$$

(A2) is expressed as

(A8) $$\begin{array}{c} V\left(G\right)\\ =E_{x∼P_{1}}\left[{\left(D^{*}\left(x\right)-c\right)}^{2}\right]+E_{x∼P_{2}}\left[{\left(D^{*}\left(x\right)-c\right)}^{2}\right], \end{array}$$

(A9) $$V\left(G\right)=\left(\begin{array}{c} E_{x∼P_{1}}\left[{\left(\frac{bP_{2}\left(x\right)+aP_{2}\left(x\right)}{P_{1}\left(x\right)+P_{2}\left(x\right)}-c\right)}^{2}\right]\\ +E_{x∼P_{2}}\left[{\left(\frac{bP_{1}\left(x\right)+aP_{2}\left(x\right)}{P_{1}\left(x\right)+P_{2}\left(x\right)}-c\right)}^{2}\right] \end{array}\right),$$

(A10) $$V\left(G\right)=\left(\begin{array}{c} \int_{x}P_{1}\left(x\right){\left(\frac{\left(b-c\right)P_{1}\left(x\right)+\left(a-c\right)P_{2}\left(x\right)}{P_{1}\left(x\right)+P_{2}\left(x\right)}\right)}^{2}dx\\ +\int_{x}p_{2}\left(x\right){\left(\frac{\left(b-c\right)P_{1}\left(x\right)+\left(a-c\right)P_{2}\left(x\right)}{P_{1}\left(x\right)+P_{2}\left(x\right)}\right)}^{2}dx \end{array}\right),$$

and

(A11) $$\begin{array}{c} V\left(G\right)\\ =\int_{x}\left(\frac{{\left(\left(b-c\right)\left(p_{1}\left(x\right)+p_{2}\left(x\right)\right)-\left(b-a\right)p_{2}\left(x\right)\right)}^{2}}{p_{1}+p_{2}}\right)dx. \end{array}$$

If we set conditions as: b−c=1, $b-a=\frac{4}{3}$, and $P_{1}\approx \frac{1}{3}P_{2}$, then $V\left(G\right)$ will converge. Therefore, (A10) is re-written as

(A12) $$\begin{array}{cc} V\left(G\right)= & \int_{x}\frac{{\left(\frac{4}{3}P_{2}\left(x\right)-\left(P_{1}\left(x\right)+P_{2}\left(x\right)\right)\right)}^{2}}{P_{1}+P_{2}}dx, \end{array}$$

(A13) $$\begin{array}{cc} = & \chi_{P}^{2}\left(P_{1}+P_{2}∥\frac{4}{3}P_{2}\right), \end{array}$$

where $\chi_{P}^{2}$ represents Pearson-$\chi^{2}$ divergence [36]. It means that when the above conditions are satisfied, $\chi^{2}$ divergence can minimize the distance between $P_{1}+P_{2}$ and $\frac{4}{3}P_{2}$. So, above equation can be expressed as

(A14) $$V\left(G\right)=\chi_{P}^{2}\left(P_{D}+3P_{G}∥4P_{G}\right).$$

If all conditions are satisfied, then $P_{D}=P_{G}$. Therefore, the optimal parameters can be defined as a=4/3, b=0, and c=1. However, since the maximum value of *D* is equal to 1, the proposed parameters are applied as

(A15) $$\begin{array}{cc} \max_{D}V\left(D\right)= & E_{I_{N}∼P_{D}\left(I_{N}\right)}\left\{{\left(D\left(I_{N}\right)\right)}^{2}\right\}+E_{I_{in}∼P_{G}\left(I_{in}\right)}\left\{{\left(D\left(HNet\left(I_{in}\right)\right)-1\right)}^{2}\right\}\\ & +E_{I_{D}∼P_{G}\left(I_{D}\right)}\left\{{\left(D\left(CNet\left(I_{D}\right)\right)-1\right)}^{2}\right\}\\ & +E_{I_{N}∼P_{G}\left(I_{N}\right)}\left\{{\left(D\left(HNet\left(I_{N}\right)\right)-1\right)}^{2}\right\}, \end{array}$$

and

(A16) $$\begin{array}{cc} \min_{G}V\left(G\right)= & E_{I_{in}∼P_{G}\left(I_{in}\right)}\left\{{\left(D\left(HNet\left(I_{in}\right)\right)-1\right)}^{2}\right\}\\ & +E_{I_{D}∼P_{G}\left(I_{D}\right)}\left\{{\left(D\left(CNet\left(I_{D}\right)\right)-1\right)}^{2}\right\}\\ & +E_{I_{N}∼P_{G}\left(I_{N}\right)}\left\{{\left(D\left(HNet\left(I_{N}\right)\right)-1\right)}^{2}\right\}. \end{array}$$

To convert the minimizing problem, (A15) can be rewritted as

(A17) $$\begin{array}{cc} \min_{D}V\left(D\right) & =E_{I_{N}∼P_{D}\left(I_{N}\right)}\left\{{\left(D\left(I_{N}\right)-1\right)}^{2}\right\}+E_{I_{in}∼P_{G}\left(I_{in}\right)}\left\{{\left(D\left(HNet\left(I_{in}\right)\right)\right)}^{2}\right\}\\ & +E_{I_{D}∼P_{G}\left(I_{D}\right)}\left\{{\left(D\left(CNet\left(I_{D}\right)\right)\right)}^{2}\right\}+E_{I_{N}∼P_{G}\left(I_{N}\right)}\left\{{\left(D\left(HNet\left(I_{N}\right)\right)\right)}^{2}\right\}. \end{array}$$
